# Preoperative Progressive Pneumoperitoneum and Botulinum Toxin A in a High-Risk Patient With Loss of Domain Inguinoscrotal Hernia

**DOI:** 10.7759/cureus.68509

**Published:** 2024-09-03

**Authors:** Stanko J Baco, Jovica Mišić, Vladan Perunicic, Milos Mitric, Sonja Đukanović

**Affiliations:** 1 General Surgery, Public Health Institution Hospital “Dr Mladen Stojanović”, Prijedor, BIH; 2 General Surgery, Saint Luke the Apostle Hospital, Doboj, BIH; 3 Surgery, General Hospital, Čačak, SRB; 4 Emergency Medicine, Public Health Institution Dom zdravlja Prijedor, Prijedor, BIH

**Keywords:** inguinoscrotal hernia, multimorbid, amyand’s hernia, tension repair, shouldice technique, botulinum toxine, progressive preoperative pneumoperitoneum, loss of domain, giant inguinal hernia

## Abstract

We present a challenging case of a loss of domain (LOD) inguinoscrotal hernia in a 77-year-old high-risk patient, successfully managed with the complementary preoperative use of progressive pneumoperitoneum (PPP) and botulinum toxin A (BTA) without complications. Giant inguinoscrotal and LOD hernias, particularly in multimorbid patients, are highly complex and require meticulous preoperative preparation. In this case, PPP was performed with ambient air and a gradual increase in insufflation volume, while BTA was injected at three points on each side, with a total dose of 300 IU. This approach facilitated a complication-free increase in abdominal cavity volume and the repositioning of chronically eventrated abdominal contents. The technique proved safe, feasible, and effective, contributing to atraumatic adhesiolysis, reduced operative time, and avoidance of more invasive surgical methods. A Shouldice pure tissue repair was performed, successfully avoiding the need for prosthetic materials.

## Introduction

Inguinal hernias are common, accounting for 75% of all abdominal wall hernias and affecting nearly 25% of men and less than 2% of women over their lifetime. Giant inguinoscrotal hernias are defined as hernias that extend below the midpoint of the inner thigh while standing [1,2]. Loss of domain (LOD) hernias are characterized by the inability to achieve simple reduction of their contents and primary fascial closure without additional reconstructive techniques or without significant risk of complications due to increased intra-abdominal pressure (IAP) [3,4]. Managing LOD hernias with chronic displacement of abdominal contents is particularly challenging, especially in multimorbid patients, defined as those with two or more chronic conditions [5].

In ventral LOD hernias, several treatment options have been proposed. The component separation technique, first reported by Albanese and popularized by Ramirez, was a mainstay for complex hernia closure [6,7]. However, due to complications and high recurrence rates, new techniques and modifications have been developed. One such technique is transversus abdominis release (TAR), a modification of the posterior component separation technique, which extends the Rives-Stoppa-Wantz method with sublay mesh placement. TAR is associated with low morbidity and better outcomes, but it is not applicable to inguinal hernias, necessitating other surgical techniques or adjunct preoperative methods [6,8,9].

Progressive pneumoperitoneum (PPP), first described by Moreno in 1947, induces abdominal wall distension by creating the necessary space for hernial sac content reduction [7]. It is an excellent adjuvant technique for the preoperative treatment of LOD hernias, particularly when combined with botulinum toxin A (BTA). BTA, also described as “chemical component separation,” induces reversible paralysis of the lateral abdominal wall muscles, allowing their elongation and facilitating hernia repair [10,11].

We present a challenging case of an LOD inguinoscrotal hernia in a high-risk patient, where the complementary preoperative use of PPP and BTA led to excellent results without serious complications. The patient experienced a successful recovery and was discharged on postoperative day 3.

## Case presentation

**Figure 1:**
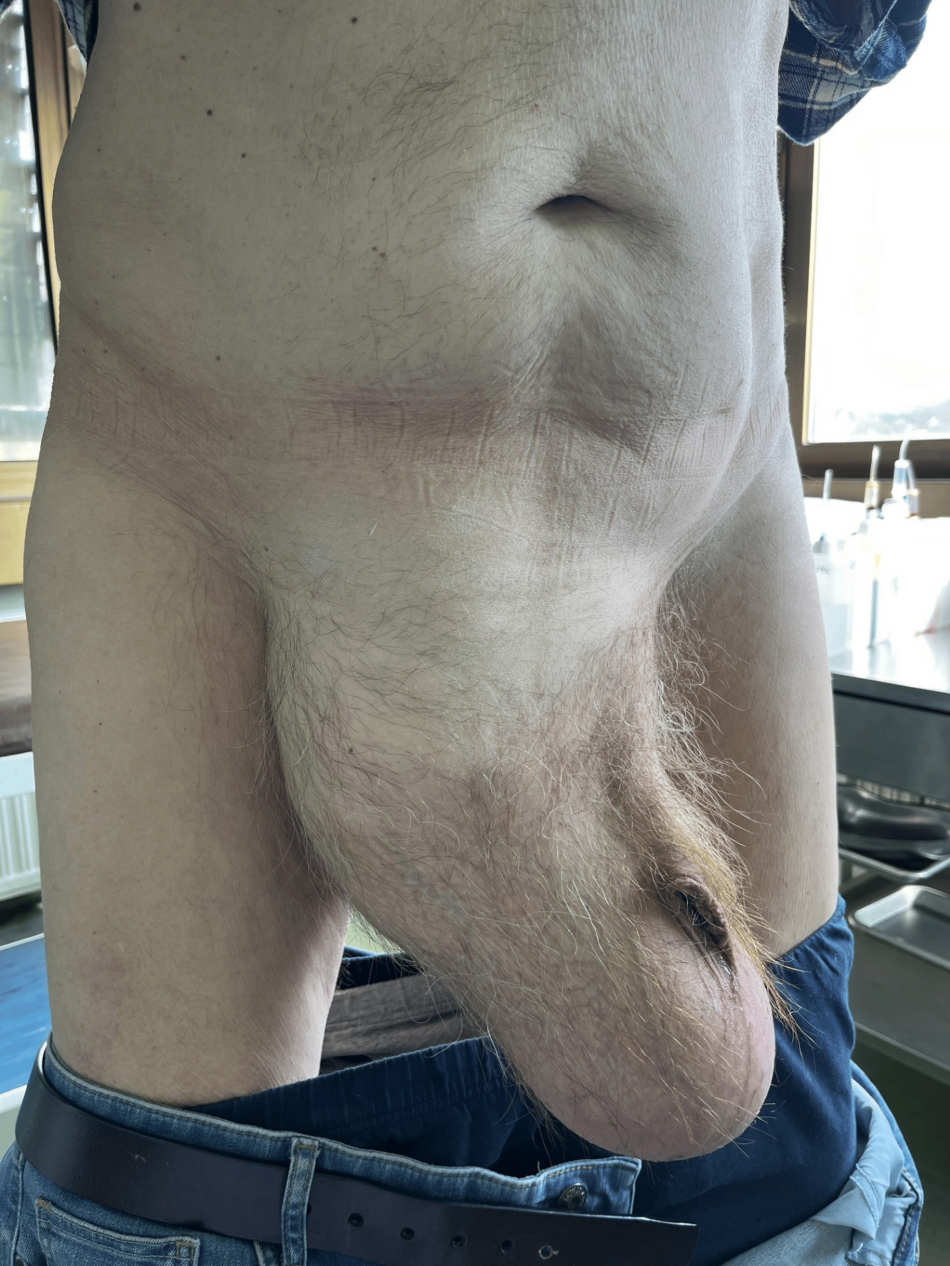
Initial examination showing a massive right inguinoscrotal hernia

**Figure 2:**
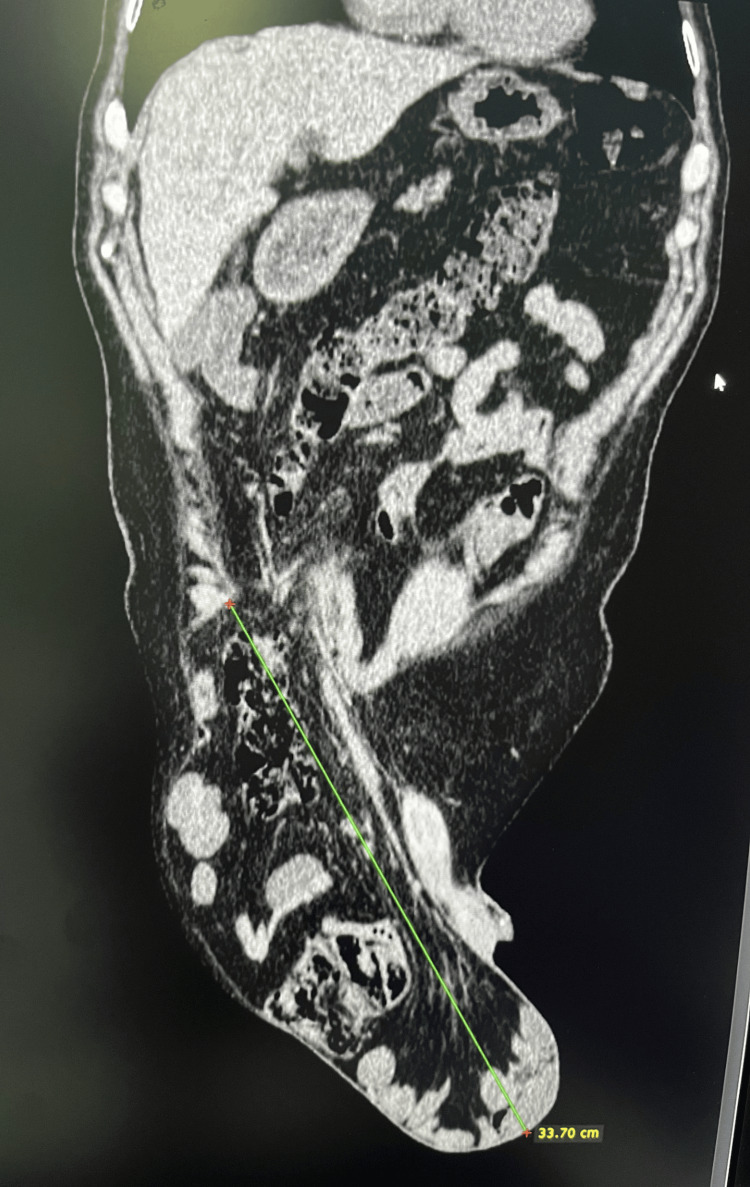
Preoperative CT scan (sagittal view) showing a 33 cm long hernial sac filled with small and large intestines, along with the mesentery

A 77-year-old man presented to our ambulance with a giant right-sided inguinoscrotal hernia that he had experienced for over 10 years (Figure 1). Due to the hernia’s enormous size, extending almost down to his knees, he suffered from abdominal and groin pain, a buried penis, difficulty with normal daily activities such as walking and dressing, and spontaneous urinary leakage. His high operative risk had led to multiple previous denials of surgery. The patient’s comorbidities included hypertension, chronic renal failure, ischemic cardiomyopathy, NYHA II heart failure, severe mitral valve insufficiency, intermittent left bundle branch block, atrioventricular block gradus primus, prostatic hyperplasia, and NSTEMI myocardial infarction three years prior. A preoperative CT scan revealed a 33 cm long hernial sac completely filled with small and large intestines (Figure 2).

**Figure 3:**
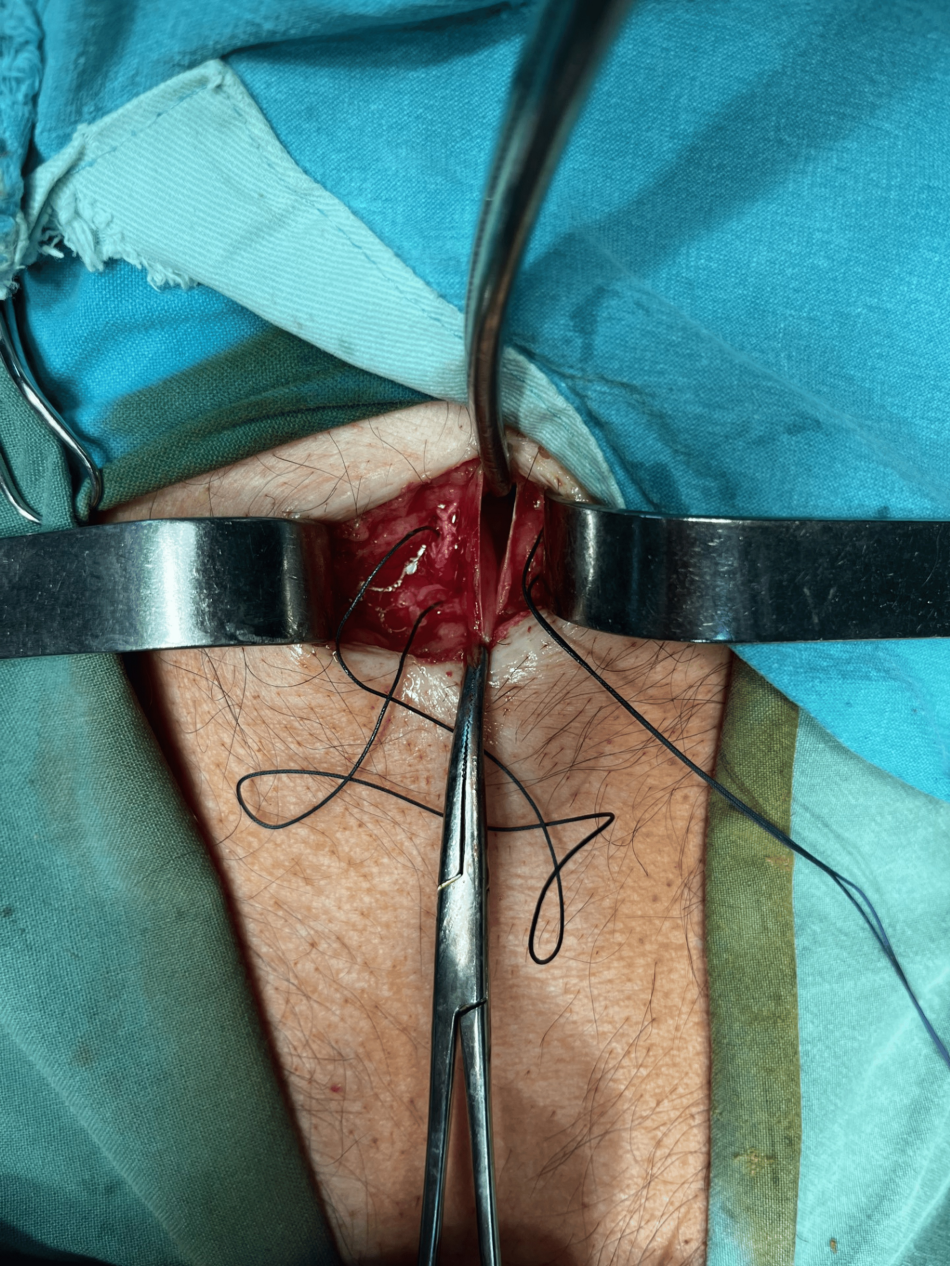
Intraoperative image of catheter placement under local anesthesia for pneumoperitoneum induction

Under local anesthesia, we placed a simple Foley catheter into the abdominal cavity through a 20-mm vertical subxiphoid incision to induce pneumoperitoneum (Figure 3). We did not use a triple valve catheter, a central venous catheter, or bacterial filters as described in other case reports. The procedure was well tolerated, and the patient was discharged on the same day with only oral analgesics.

Initially, we insufflated 600 cc of ambient air daily for the first three days, followed by ambulatory insufflation of 700 cc per day for the next seven days. IAP was not monitored. A surgical site infection occurred due to irregular wound dressing, which was resolved with daily cleansing using water and soap. The abdominal distention caused only mild discomfort after each insufflation.

**Figure 4:**
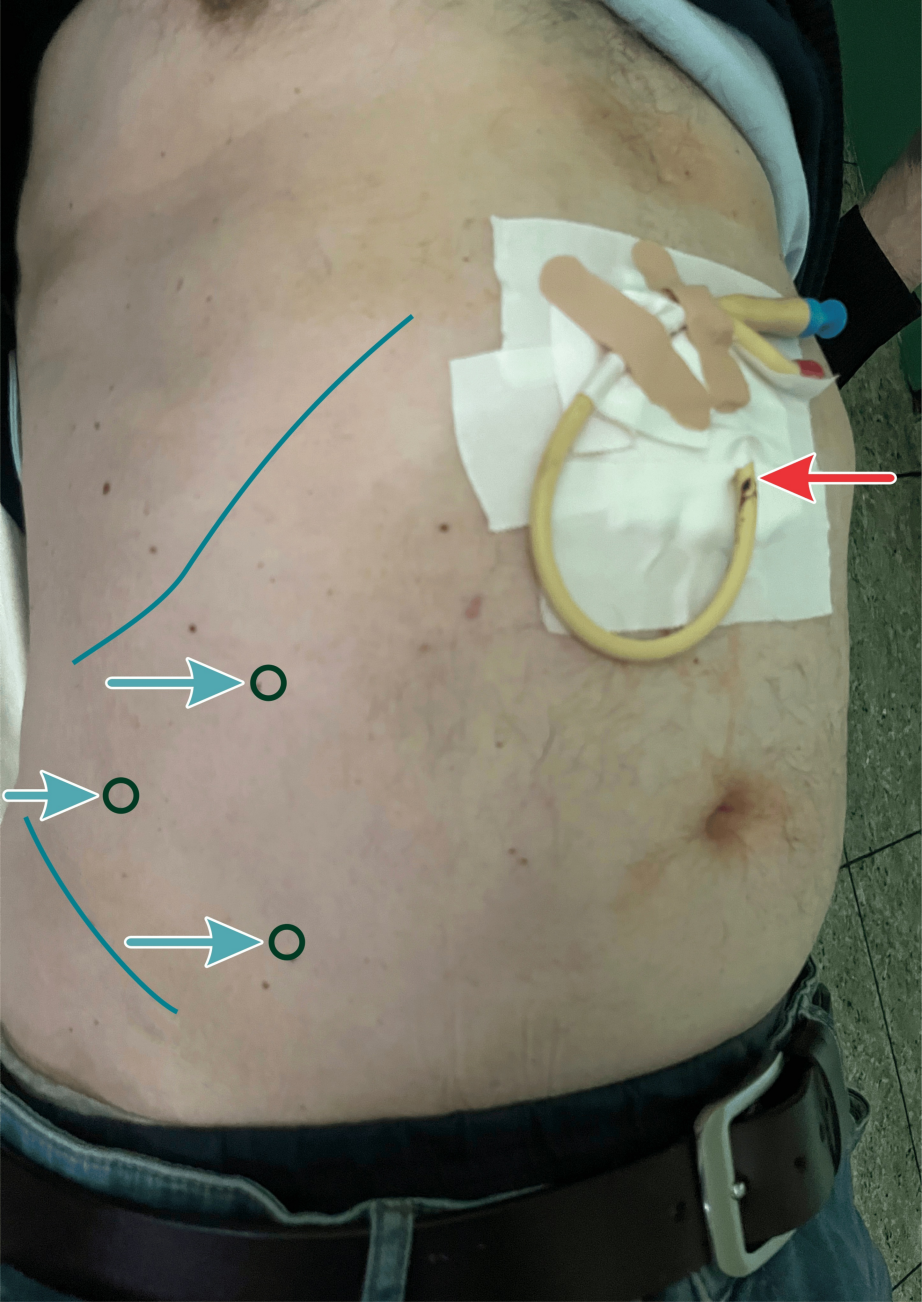
BTA injection points are located between the costal margin, iliac crest, and lateral to the semilunar line The red arrow indicates the placement of the intraperitoneal Foley catheter.

After 10 days and an insufflated volume of 6.7 L, we were not satisfied with the progress of abdominal wall distention, so we proposed BTA to the patient. We injected 300 IU of BTA between the external and internal oblique muscles, 150 IU on each side of the abdominal wall at three points between the costal margin, iliac crest, and lateral to the semilunar line. The procedure was performed under ultrasound guidance and with informed consent (Figure 4).

Two weeks after the BTA injection, we conducted a follow-up abdominal CT scan. The patient continued with daily self-sufflation at home. Five days before surgery, the patient was admitted to the hospital and underwent insufflation with 1,000 cc of room air three times and then 1,400 cc twice. The total insufflation period was 20 days, with daily volumes ranging from 600 to 1400 cc, amounting to a total of 20.7 L of ambient air.

**Figure 5:**
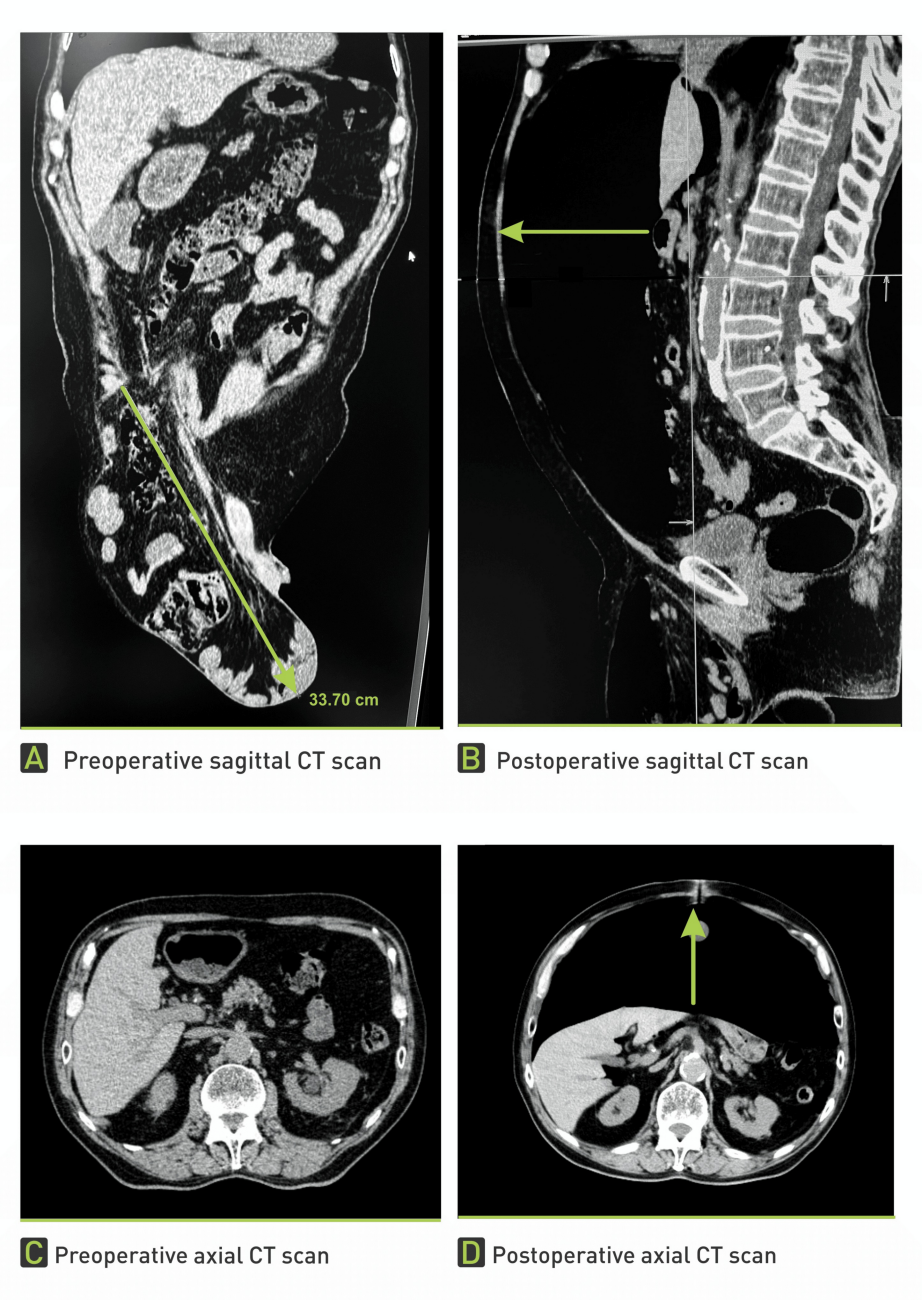
Control CT scan showing a noticeable enlargement of the abdominal cavity (green arrows) and sufficient space created for herniated viscera

Following BTA application and the increased insufflation volume, the patient experienced more pronounced abdominal and groin pain and distention, particularly when standing. The abdomen was noticeably more distended. A control CT scan revealed an enlargement of the abdominal cavity, providing sufficient space for the herniated viscera (Figure 5).

**Figure 6:**
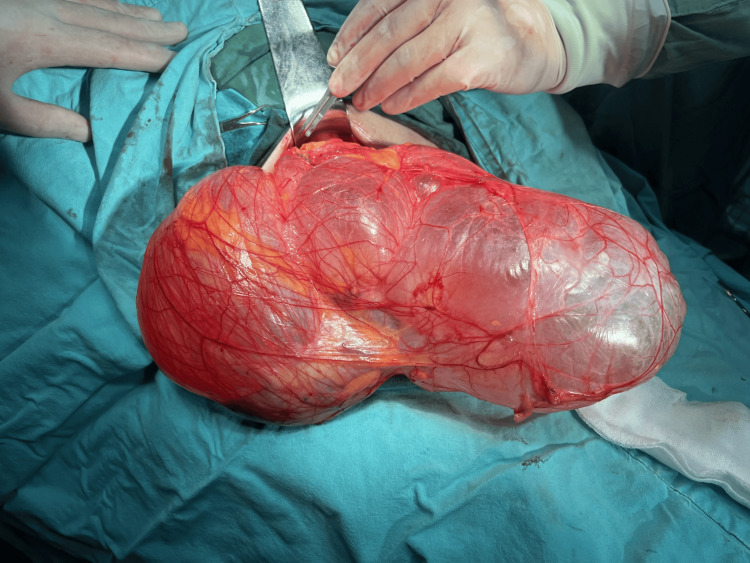
Intraoperative image of the hernial sac prepared and filled with air

**Figure 7:**
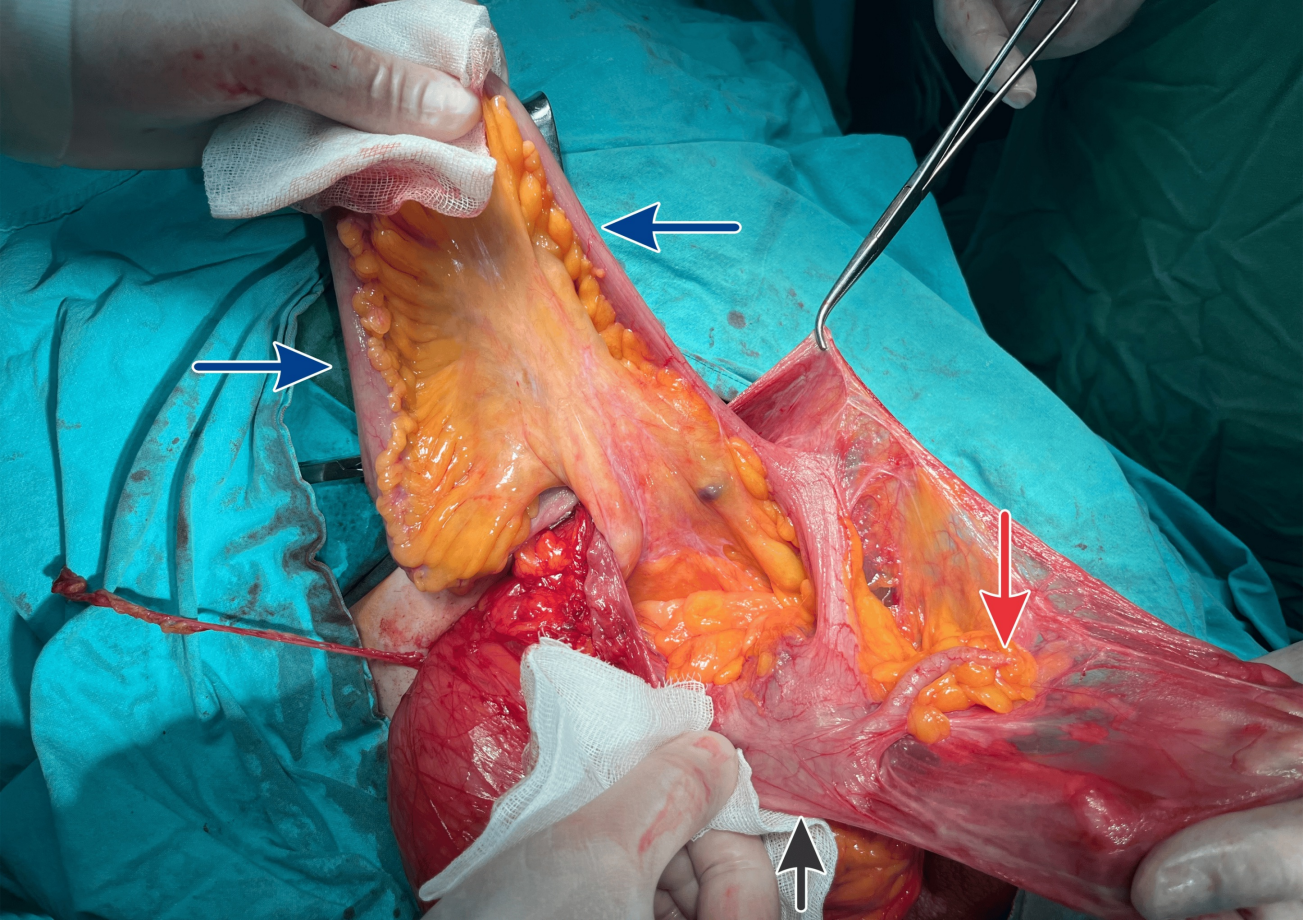
Hernial sac after partial reduction of herniated contents, showing the small bowel (blue arrows), cecum (black arrow), and appendix (red arrow), indicating an Amyand’s hernia

**Figure 8:**
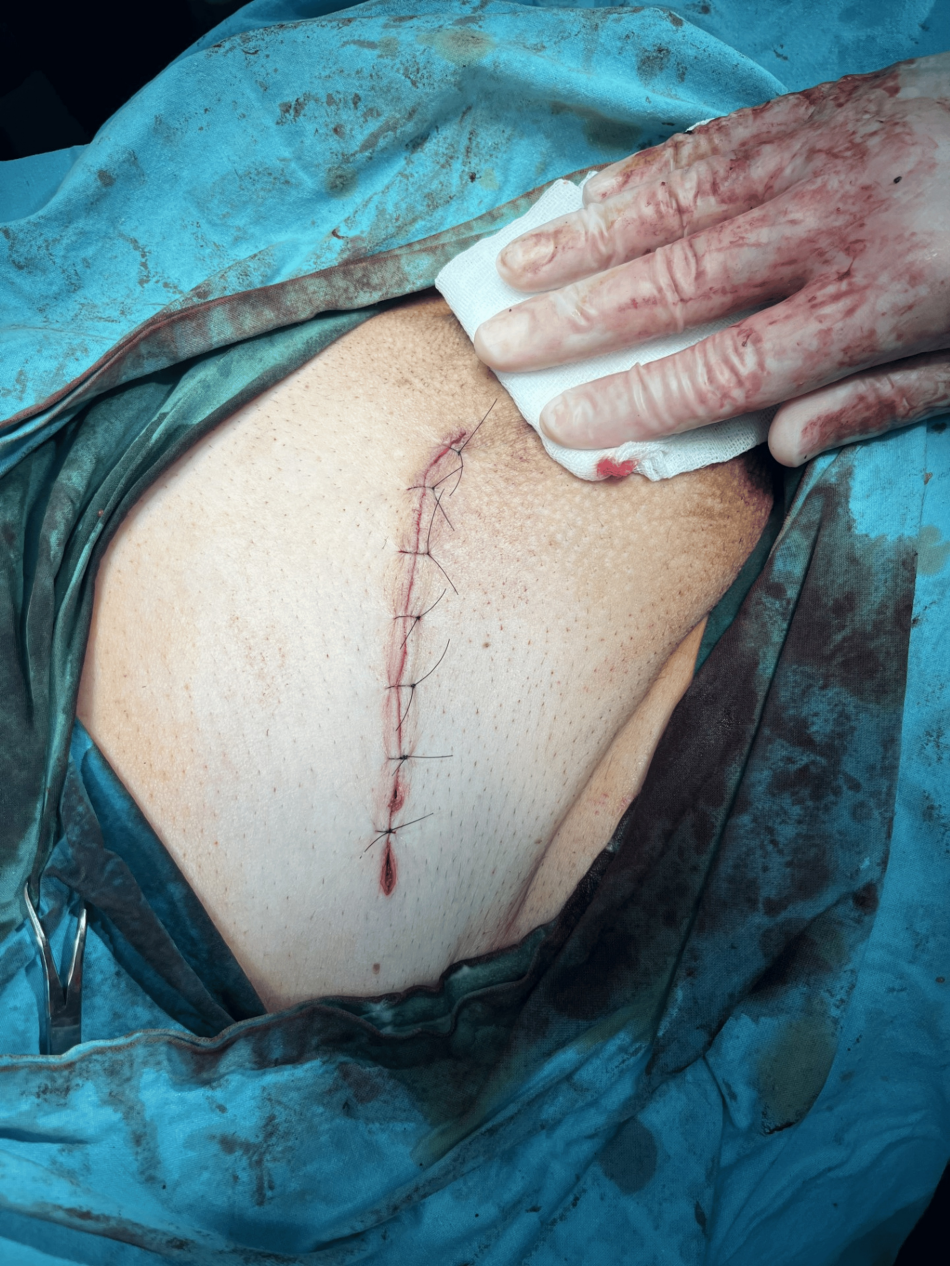
Immediate postoperative image showing the outcome of the Shouldice repair

Perioperatively, 30 minutes before surgery, the patient received 1 g of cefazolin, 0.5 g of metronidazole, and deep vein thrombosis prophylaxis. During the operation, we opened the hernia sac, revealing an appendix, colon ascendens, and numerous small bowel loops (Figure 6, Figure 7). We restored the hernia contents without the need for bowel resection and performed a Shouldice natural tissue repair (Figure 8). The surgery lasted 90 minutes.

**Figure 9:**
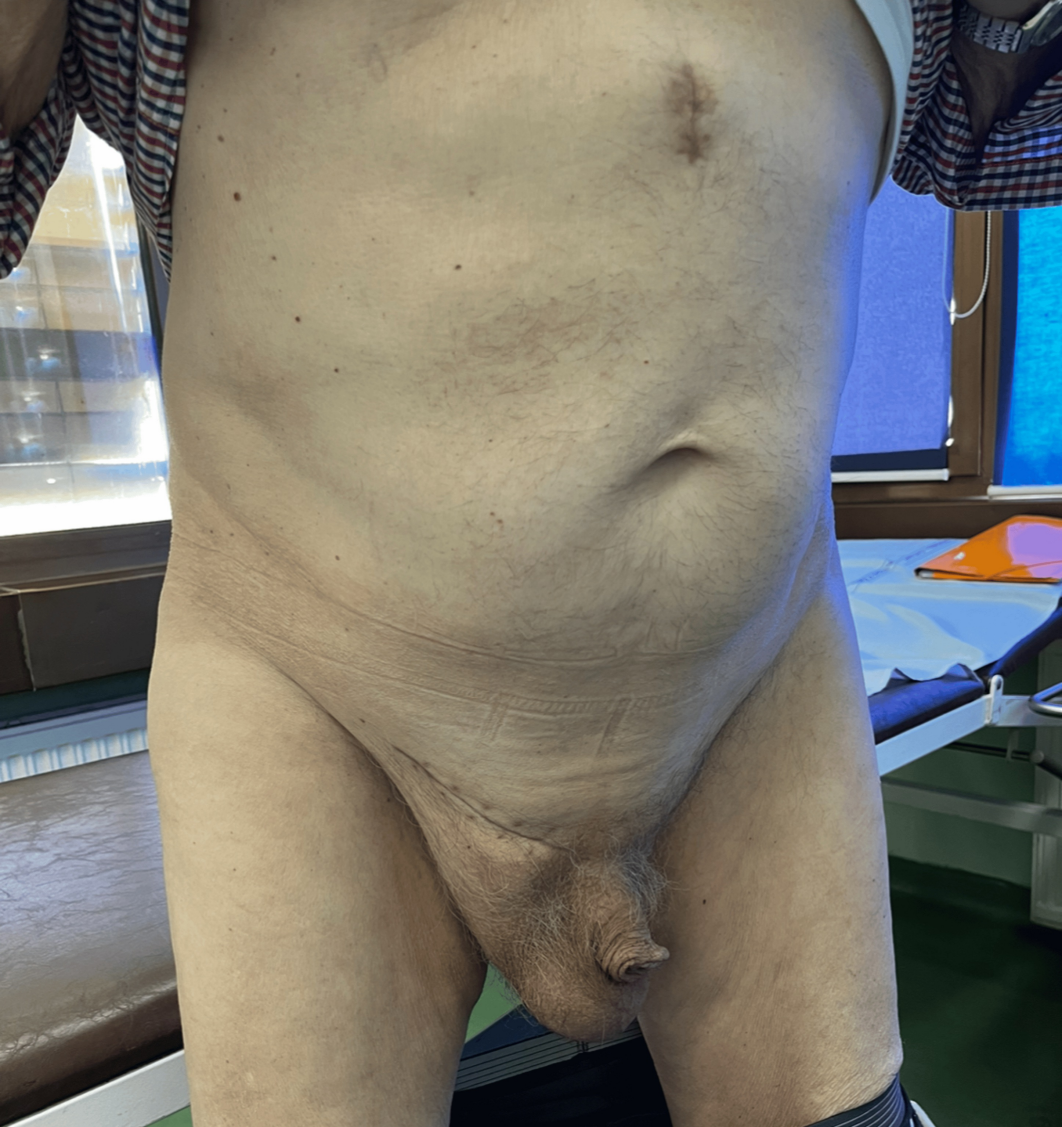
Image of the surgical site six months postoperation

The patient’s recovery proceeded smoothly, and they were discharged on the 10th postoperative day. During the first outpatient follow-up, an inguinal wound hematoma was noted after seven days, which resolved spontaneously. Five months postoperation, there is no sign of recurrence, and the patient reports high satisfaction with their quality of life (Figure 9).

## Discussion

The classification of hernias has seen various proposals, aiming to enhance the utility of LOD as an outcome predictor and standardize morphological descriptions. International specialists have reached a consensus on defining LOD hernias to minimize clinical inconsistencies. This definition specifies that LOD hernias either pose a significant risk of complications due to increased IAP or require additional reconstructive procedures beyond primary fascial closure and simple content reduction [4]. An alternative definition considers hernias with more than 20% of abdominal contents as straightforward [12]. There is ongoing debate about the distinction between reversible and irreversible LOD hernias, with no clear cutoff point [4].

Managing LOD hernias, which involve chronic displacement of abdominal contents, is particularly challenging. These high-risk operations should be performed by experienced hernia specialists, especially in multimorbid patients, as multimorbidity significantly impacts functioning and survival, contributing to nearly 70% of postoperative deaths [13]. PPP is a prehabilitation technique that can expand the abdominal cavity, creating space for chronically impacted contents and reducing the risk of compartment syndrome and related complications. The PPP catheter placement procedure is reproducible even in low-resource settings, using ambient air and local anesthesia. While some studies have used CO2 for PPP creation, its rapid resorption makes it less suitable [14]. Our experience showed no complications from room air insufflation. However, PPP alone initially did not yield satisfactory results, and we observed discomfort when the injected air volume exceeded 1,000 cc, suggesting a gradual approach is preferable. Prolonged PPP can lead to effective pneumatic adhesiolysis, and IAP measurement is not obligatory.

BTA, known for its use in treating hyperhidrosis and wrinkles by paralyzing muscles, also reduces abdominal wall tension in hernia surgery [15]. BTA allows for reversible separation of abdominal wall components, reducing the need for invasive surgery and associated complications. Our experience with BTA, administered at three points on each side with a dose of 300 IU, showed satisfactory results. BTA’s maximum effect appears after 10-14 days and lasts approximately three months [16,17]. We opted not to use mesh implantation or drains, based on the patient’s health status, local findings, and the operator’s expertise, with very satisfactory results from the Shouldice operation. Studies indicate a recurrence rate below 1% with the Shouldice technique, which is particularly advantageous in low-resource settings [18]. In our case, the combined use of PPP and BTA achieved pneumatic adhesions, dissection of the hernial sac, and a significant reduction in operative time. This approach is crucial for multimorbid patients, avoiding more invasive techniques such as component separation, small bowel resection, or hemicolectomies.

## Conclusions

Giant inguinoscrotal and LOD hernias, especially in multimorbid patients, require preoperative prehabilitation. In our experience, PPP alone was insufficient for optimal conditions. However, when combined with BTA, we achieved excellent results. This approach is safe, feasible, and effective, facilitating pneumatic, nontraumatic adhesiolysis, reducing surgery time, and minimizing the need for more invasive techniques such as hemicolectomies, component separation, or TAR. The catheter placement procedure is reproducible even in low-resource settings and can be performed under local anesthesia. It is crucial to close the fascia with a non-resorbable suture for pneumoperitoneum creation and maintain regular wound care. We found that a BTA injection at three points on each side with a total dose of 300 IU was sufficient. Due to notable discomfort with injected volumes exceeding 1000 cc per day, we recommend a gradual and slower increase in volume over an extended period.
